# Isolation and characterization of diverse microbial representatives from the human skin microbiome

**DOI:** 10.1186/s40168-020-00831-y

**Published:** 2020-04-22

**Authors:** Collin M. Timm, Kristin Loomis, William Stone, Thomas Mehoke, Bryan Brensinger, Matthew Pellicore, Phillip P.A. Staniczenko, Curtisha Charles, Seema Nayak, David K. Karig

**Affiliations:** 1 Research and Exploratory Development Department, Johns Hopkins University Applied Physics Laboratory, Laurel, MD USA; 2 Brooklyn College, City University of New York, Brooklyn, NY USA; 3 Division of Infectious Diseases, Department of Medicine, Johns Hopkins University School of Medicine, Baltimore, MD USA; 4 Department of Bioengineering, Clemson University, Clemson, SC USA

**Keywords:** Skin microbiome, Isolate collection, Carbon source utilization

## Abstract

**Background:**

The skin micro-environment varies across the body, but all sites are host to microorganisms that can impact skin health. Some of these organisms are true commensals which colonize a unique niche on the skin, while open exposure of the skin to the environment also results in the transient presence of diverse microbes with unknown influences on skin health. Culture-based studies of skin microbiota suggest that skin microbes can affect skin properties, immune responses, pathogen growth, and wound healing.

**Results:**

In this work, we greatly expanded the diversity of available commensal organisms by collecting > 800 organisms from 3 body sites of 17 individuals. Our collection includes > 30 bacterial genera and 14 fungal genera, with *Staphylococcus* and *Micrococcus* as the most prevalent isolates. We characterized a subset of skin isolates for the utilization of carbon compounds found on the skin surface. We observed that members of the skin microbiota have the capacity to metabolize amino acids, steroids, lipids, and sugars, as well as compounds originating from personal care products.

**Conclusions:**

This collection is a resource that will support skin microbiome research with the potential for discovery of novel small molecules, development of novel therapeutics, and insight into the metabolic activities of the skin microbiota. We believe this unique resource will inform skin microbiome management to benefit skin health.

Video abstract.

## Background

Skin is a constantly growing and changing barrier tissue that acts as a first line of defense against many environmental factors. The skin micro-environment varies across the body, but all sites are host to microorganisms that can affect the maintenance and disruption of skin health. Some of these organisms are true commensals which colonize a unique niche on the skin, while open exposure to the environment results in transient colonization by diverse microbes with an unknown contribution to skin function. Culture-based studies of skin microbiota suggest that skin microbes can affect skin properties [1], immune responses [2, 3], pathogen growth [4], wound healing [5], and even disease vector attraction [6].

The skin microbiome has been studied by both culturing and sequencing, and *Actinobacteria*, *Firmicutes*, *Bacteriodetes*, and *Proteobacteria* have been identified as major dominating phyla [7]. The proportion of organisms within these groups changes by body site, with sebaceous (oily) sites such as the forehead represented by *Cutibacterium*, *Staphylococci*, and *Corynebacteria*. Moist sites such as the antecubital fossa (inner elbow) are dominated by *Proteobacteria* and *Staphylococci*, and dry sites such as the forearm or legs being the most diverse but primarily dominated by *Corynebacteria*, *Flavobacteriales*, or β-*Proteobacteria* [8]. A number of factors likely influence the microbiome composition at different body sites. These factors include moisture content, temperature, pH, and the resources available for microorganism metabolism at each site. Sequencing studies have associated bacterial genes in skin swabs with metabolic pathways for compounds present on the skin such as sugars, lipids, and iron [9]. Some bacteria have been found to co-localize at body locations with specific skin residual compounds. For example, *Cutibacterium* is enriched at sites with compounds such as oleic acid and palmitic acid [10]. Similarly, the use of personal care products that introduce additional compounds to the skin surface has been found to alter the microbiome composition [11].

For decades, culture-based studies were conducted to characterize the bacterial flora of the skin, probing differences among body sites, effects of hygiene and behavior, and roles in disease [12]. While revealing key insights, these studies were limited by the ability to culture diverse microbes from the skin. DNA sequencing has since driven far more detailed characterizations. For example, in chronic wound studies, molecular testing was compared to culture-based methods, and results showed that molecular techniques identified 85% of cultured representatives, while culturing only identified 16% of genera detected by sequencing [13]. Nonetheless, while offering powerful sensitivity, sequencing information is also associated with various forms of error and bias. Reagent contamination and imperfect sampling conditions can generate false positives, particularly due to the low input nature of skin swabs [14, 15]. In addition to these error sources, bias can be introduced during DNA extraction, as efficient disruption of all taxa in a diverse sample is challenging. For amplicon sequencing, bias can also result from primer mismatches and deviations from balanced GC-content [16].

Perhaps more importantly, insights gained from sequencing-based studies are based on correlations, often without definitive identification of the causative relationships between taxa/genes and function. Thus, regardless of the new wealth of information on the spatial ecology of the skin microbiome [8], its temporal stability [17], its variation across individuals [18], and its response to disruption [19, 20], culture-based bottom-up studies will remain important for revealing mechanistic explanations of results stemming from sequence-based top-down measurements [21]. These mechanistic explanations will be essential for ultimately tapping into the therapeutic potential of microbiome manipulation.

Indeed, cultured isolates have offered tremendous value to the study of multiple microbiome systems [12]. In the gut microbiome, cultured representatives have contributed to our understanding of vitamin production by probiotic organisms [22], microbiome structure including adhesion [23], the chemical modification and metabolism of drugs [24], and the tracking of microbes [25]. In plant environments, bacterial strains were used to show synergistic effects of microbiome members in complex communities [26]. On the skin, cultured microorganisms have been shown to activate TLR2 and stimulate the immune response, leading to increased resistance to skin pathogens [3]. Cultured isolates from skin have also been used to study VOC profile modification, which could lead to strategies of skin microbiome management that ultimately affect vector attraction [27]. More conclusively, culture-based studies have shown that *S. epidermidis*, when cultured and re-applied to its original human host, can inhibit the growth of *S. aureus* in a skin microbiome [4].

Recognizing the need for a diverse and controlled set of commensal isolates to study the skin microbiome, we collected samples from 17 diverse, healthy research participants. Organisms were collected from a dry site, the forearm (AM), a moist site, the antecubital fossa (AF), and a sebaceous site, the forehead (FM), from each individual by swabbing and aerobic isolation on blood agar. For four of the 17 participants, additional growth conditions were used, including anaerobic cultures, potato dextrose agar for fungi [28], low nutrient agar to capture slow-growing organisms [29], and spore treatments. The isolates described here have been cataloged and stored for future research, and include accompanying survey data on participant history and skin functional data from isolation sites. To showcase the utility of this collection and gain insight into the functional capabilities of skin microbiome constituents, we characterized a subset of isolates for their ability to metabolize skin surface compounds. While we found some phylogenetic patterns in metabolic abilities, we also observed significant variations across taxa within the same genus, thus drawing attention to the need for analyzing functional activity at the species or strain strain-level. This characterization effort exemplifies one of many possible applications of the isolates collection to gain information and insights into the forces shaping skin microbial communities.

## Results

### Isolation conditions and counts

**Fig. 1:**
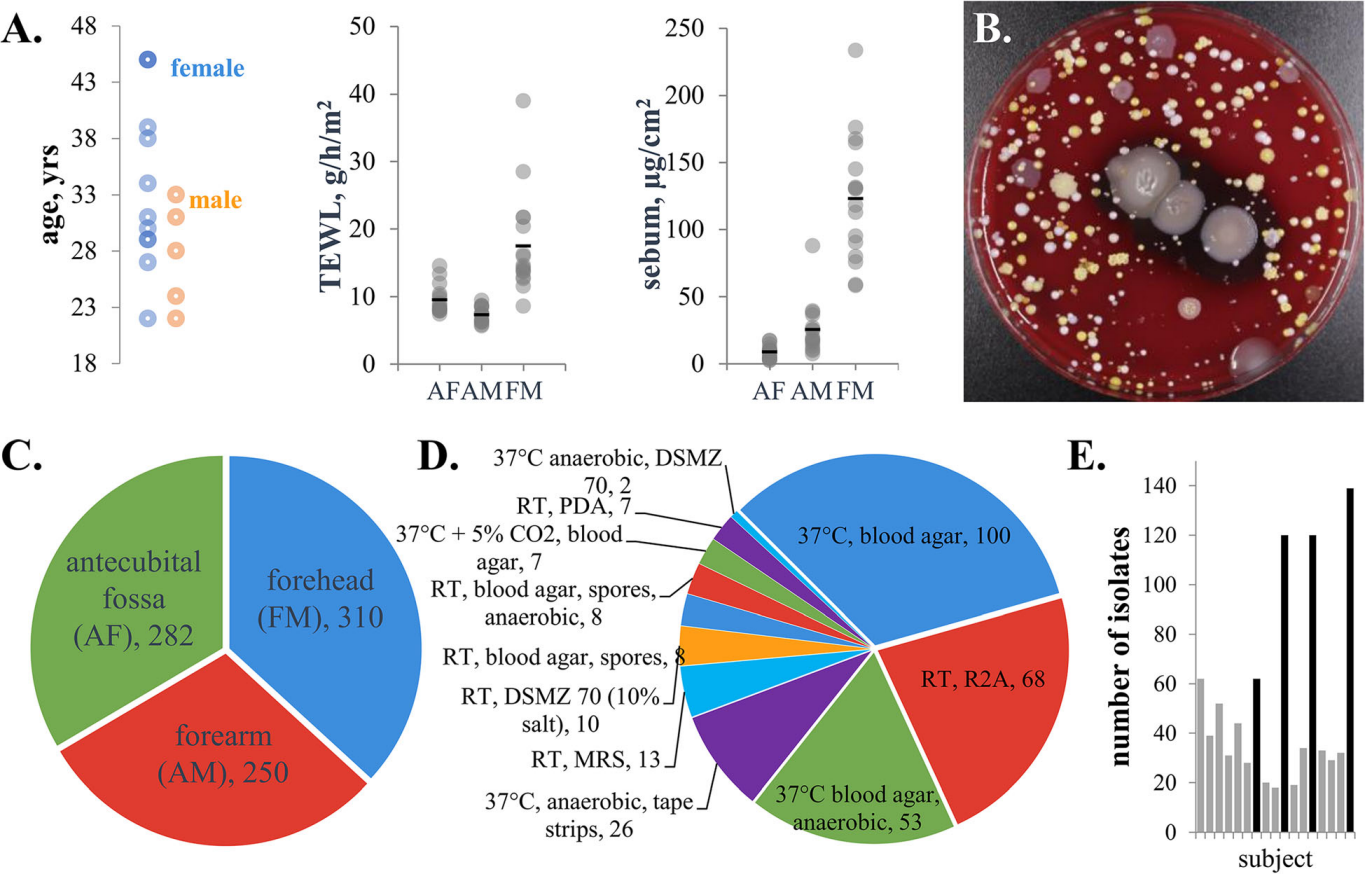
Summary of isolation sites and conditions. **a** Research participants metadata includes age, gender, trans-epidermal water loss, and sebum content. **b** Example blood agar plate showing colony morphologies. **c** Total number of isolates by site. **d** Isolate counts from growth conditions, not including the standard condition of room temperature (RT), blood agar. **e** Total number of isolates by research participant. Black bars signify research participants for which multiple isolation conditions were performed. See Table S1 for additional metadata

We isolated microbial strains from the forehead, forearm, and antecubital fossa (inner elbow) to represent sebaceous, dry, and moist sites from 17 healthy volunteers. Volunteers ranged in age from 22 to 45 with 5 males and 12 females. Metadata collected includes ethnicity, country of birth, travel patterns, diet, and site-specific measures including transepidermal water loss and sebum content (Fig. 1a). Using 12 unique growth conditions, we collected 842 strains. Colonies were picked based on phenotypic diversity (representative plate shown in Fig. 1b) with 310 strains from the forehead, 250 from the forearm, and 282 from the antecubital fossa (Fig. 1c). From 4 of the 17 participants, we isolated strains from a total of 12 unique growth conditions. After our baseline isolation (blood agar, room temperature), blood agar incubated at 37 °C provided the most isolates (100), with R2A media and anaerobic culture conditions providing 68 and 52 isolates, respectively (Fig. 1d). This strategy yielded between 18 and 139 distinct isolates between participants (Fig. 1e).

### 16S identification of isolates

**Fig. 2:**
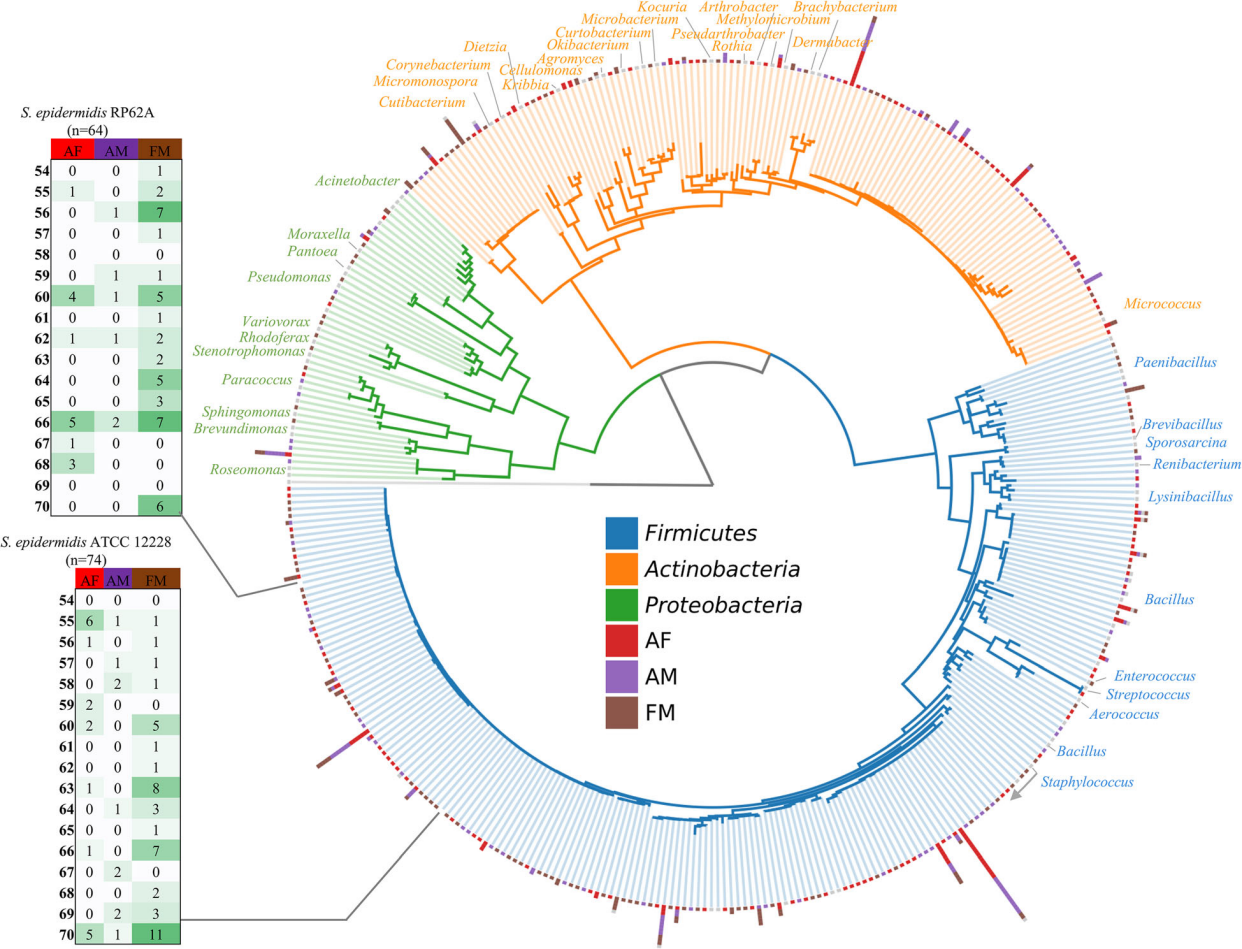
Phylogenetic tree of bacterial isolates. 16S tree for bacterial isolates. Sequences with 99% similarity are collapsed into a single node. Concentric columns indicate number of 99% similar 16S sequences by color-coded site. Pop-out tables indicate number of isolates by research participants/site that map to common *S. epidermidis* strains

Trimmed ribosomal sequences were used to generate a phylogenetic tree. Figure 2 shows the result of this tree including 83 reference strains identified as the best BLAST hit to 100% identical sequence clusters (Additional file 1 Table S4). The collection is dominated by *Firmicutes*, with the majority of isolates in the *Staphylococcus* genus (*n* = 398) and high representation of the *Bacilli* (*n* = 66). The pop-out tables show the two largest groups, both of which had perfect 16S match to *S. epidermidis* ATCC 12228 or RP62A. These results from the *Staphylococcus* groups show that all research participants had at least one *Staphylococcus* representative, and that many of the strains had identical 16S sequences between research participants and sites. Our collection also has a high representation of *Actinobacteria*, dominated by the *Micrococcus* genus (*n* = 104). Interestingly, we observe 47 *Micrococcus* isolates from the antecubital fossa, 41 from the forearm, and only 13 from the forehead. Also within the *Actinobacteria*, we have a large representation of the *Cutibacterium* genus (*n* = 33), all isolated using anaerobic isolation culture conditions, with the majority of these isolated from the forehead (22 foreheads, 6 antecubital fossa, 5 forearms). These organisms are most closely related to *Cutibacterium acnes*, recently renamed from the *Propionibacterium acnes* group [30]. The *Proteobacteria*phylum includes diverse representatives that are rarer in our collection. The *Acinetobacter* genus has 7 isolates from 3 research participants, while the *Roseomonas* genus has 13 isolates from 4 research participants with 8 of the strains isolated from the forearm. Of the 83 groups collapsed by best BLAST hit (Additional file 1 Table S4), 43 contained only a single isolate. Of the remaining 40 groups made up of multiple isolates, only 2 groups contained all isolates from the same research participant site: a group most closely related to *Paenibacillus qingshengii* from the forehead of research participants 066 and a group most closely placed in the *Variovorax* genus also from research participant 066. This result suggests that the remaining 38 groups include isolates from multiple participants/sites that would be interesting for strain variability studies.

### Collection diversity

**Fig. 3:**
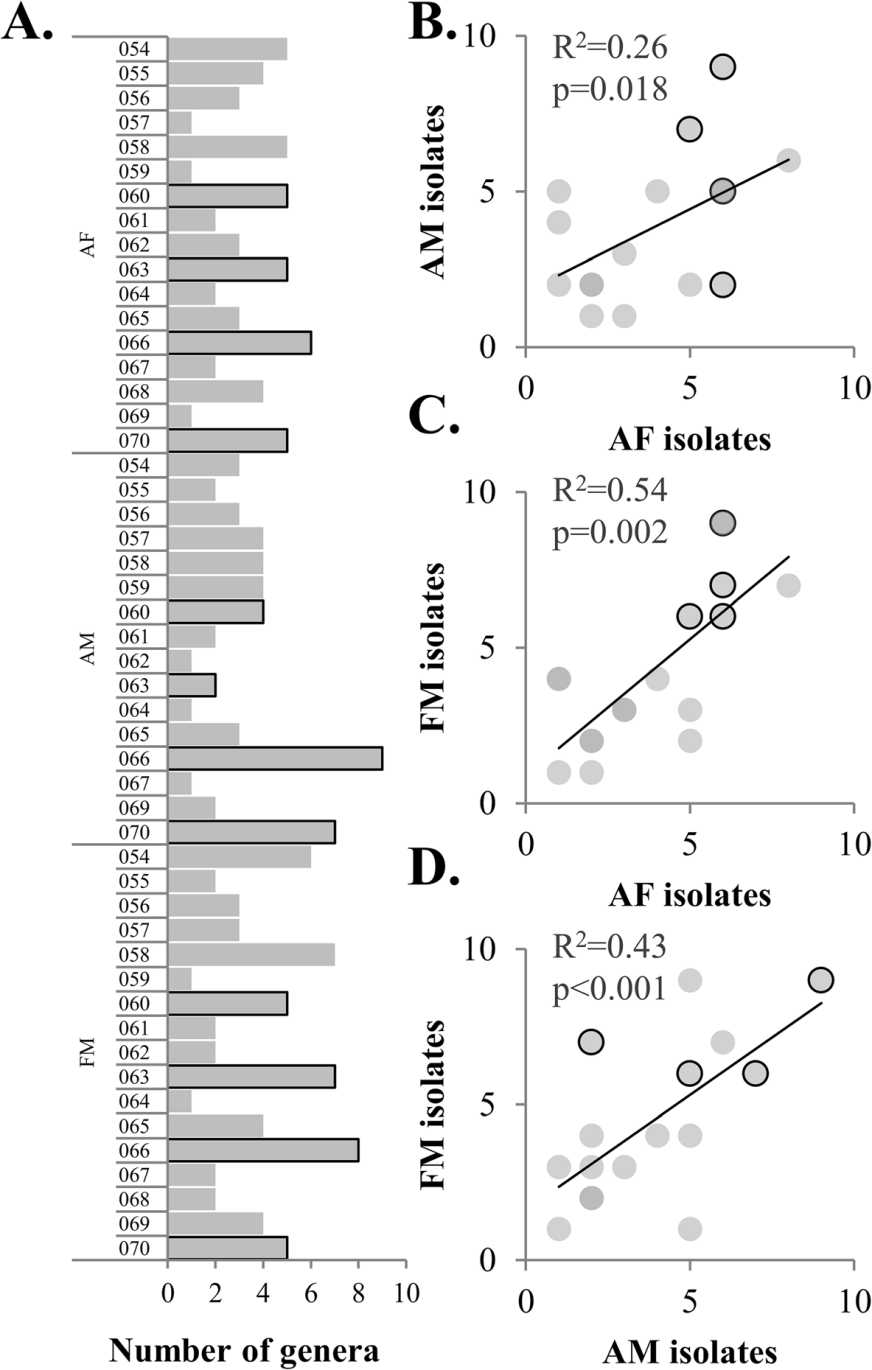
Bacterial diversity between subjects/sites. Correlation between number of genera by site (raw data shown in **a** for **b** antecubital fossa (AF) vs. forearm (AM), **c** antecubital fossa (AF) vs forehead (FM), and **d** forearm (AM) vs. forehead (FM). *p* values were calculated from Pearson correlation with 17 pairs. Outlined bars and points indicate subjects with multiple isolation conditions

The number of unique genera identified by culturing ranged from 1 to 12 for any given participant/site combination, with a total of 40 genera collected (Additional file 1 Table S3). While the research participants with the highest number of unique genera included multiple isolation conditions, the research participants with the second and third most genera had isolates collected only from blood agar, not the additional growth conditions described above. First, our in-depth sampling of four research participants (using diverse media/growth conditions) resulted in increased diversity from those research participants (> 16 vs. 9 genera, *p* < 0.01, Student’s *t* test). Interestingly, we observed a trend between a number of isolates from sites within a single research participant, suggesting that diversity across the cultivable fraction of the microbiome is conserved (Fig. 3). Notable groups include isolates from the *Micrococcus* genus which was highly represented in the isolate collection but has been observed at low abundance in published amplicon data for the skin microbiome. Similarly, our collection includes 12 *Paenibacillus*, 8 *Roseomonas*, and 7 *Dietzia* isolates distributed across multiple individuals, suggesting a role of these organisms in some skin communities (Additional file 1 Table S7). Additionally, we investigated the resulting diversity from our chosen isolation conditions and observed that after room temperature blood agar conditions, anaerobic blood agar, R2A, and tape strips yielded the greatest additionally diversity of isolates (Table S8).

### Fungal isolates

**Fig. 4:**
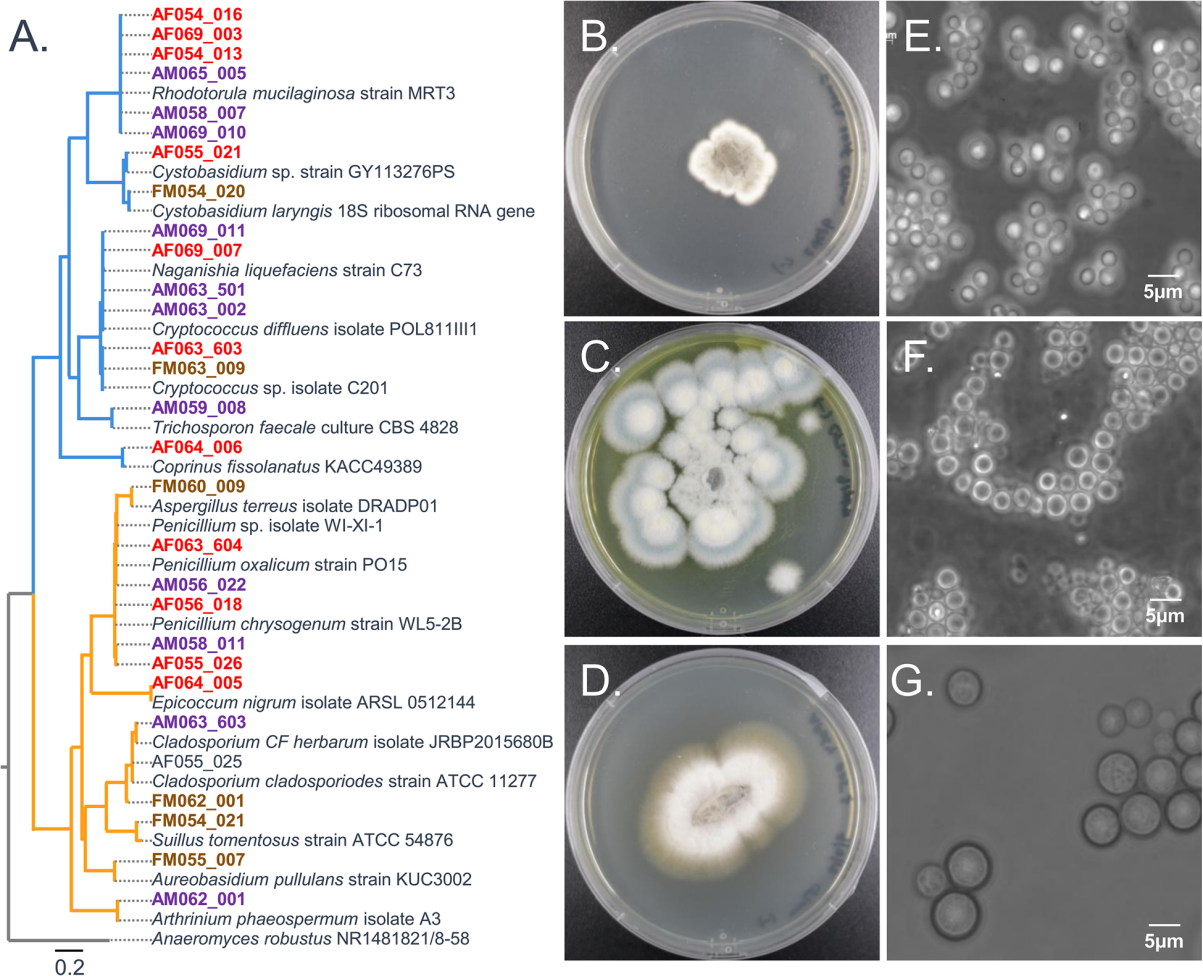
Fungal isolates. **a** ITS tree. Blue branches are *Ascomycota* and orange branches are *Basidiomycota*. **b** Isolate FM062_001 identified as *Cladosporium caldospoiroides*. **c** Isolate AF056_018 identified as *Penicillium dipodomyicola*. **d** Isolate AF064_005 identified as *Epicoccum nigrum*. **e** Isolate AF069_007 identified as *Naganishia liquefaciens*. **f** Isolate AM063_501 identified as *Naganishia liquefaciens*. **g** Isolate AF054_013 identified as *Rhodotorula mucilaginosa*

In addition to bacteria cultures which were the primary target of this study, 24 fungal isolates were collected from these samples. Fungal isolates were identified as mold growth on plates, or by microscopic examination. Fungal isolates were identified by ITS sequencing and represent taxa that are commonly found on the skin or indoor environments. For example, isolate FM063_009 closely aligned with the genus *Naganishia* which has representatives found on human skin [31] or in indoor environments [32]. A tree was built based on the ITS regions and shows the distribution of these organisms (Fig. 4, Table S5). Selected images of fungal plates show growth and yeast morphology.

### Carbon source utilization for selected strains

**Fig. 5:**
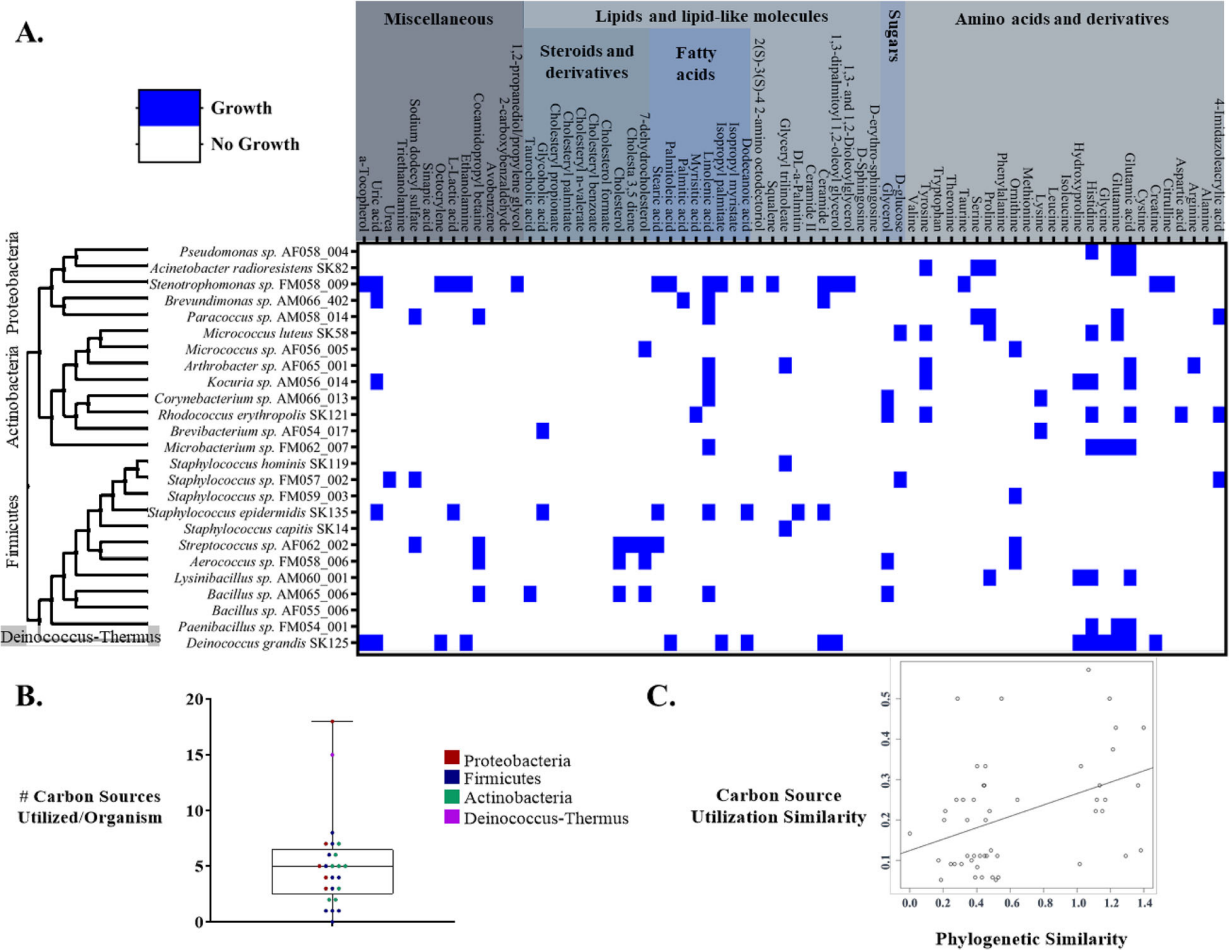
Skin resource utilization by skin microbiota isolates. **a** Resource utilization screen. Blue cells indicate a significant color change by the reduction-reporting dye; white cells indicate no significant color change not observed. **b** Scatterplot of the number of resources found to be utilized by each organism. **c** Carbon source utilization for generalists across skin isolates is positively related to phylogenetic similarity (four or more carbon sources *R*^*2*^ = 0.169; *p* = 0.003)

A subset of microbiota isolates as well as several publicly available reference strains were assayed for the ability to reduce a tetrazolium-based dye, indicating metabolic activity, in the presence of skin compounds as the sole carbon sources. The assay detected utilization for a variety of compound types, including amino acids, steroids, lipids, and molecules sourced from personal care products, such as octocrylene, a common ingredient in sunscreens (Fig. 5a). The assay also indicates clear differential metabolic capabilities across bacteria that are phylogenetically similar. For example, the two *Micrococcus* isolates did not share the capacity to utilize any of the same carbon sources. Overall, bacteria were found to utilize a range of numbers of carbon sources present on the skin (Fig. 5b). We used this approach to examine the relationship between phylogeny and carbon source utilization in the skin environment. Phylogenetic distance analysis was performed on 24 microbial taxa and 58 carbon sources. Mantel tests and linear regression models were run on all taxa together, as well as a subset of taxa classed as generalists that utilized four or more carbon sources. The Mantel test for all taxa was not significant (*R*^*2*^ = 0.000; *p* = 0.357). The linear regression model including all taxon pairs was also not significant (*R*^*2*^ = 0.000; *p* = 0.763). However, results for generalists only, defined as taxa using four or more carbon sources, were all significant (Fig. 5c): Mantel test (*R*^*2*^ = 0.349; *p* = 0.009); linear regression model including all taxon pairs (*R*^*2*^ = 0.122; *p* = 0.002). Results were qualitatively similar when generalists were defined as utilizing five or more carbon sources.

## Discussion

In this study, we isolated and characterized over 800 organisms from 3 body sites on 17 healthy research participants. We believe that the controlled isolation sites, paired with in-depth participant metadata, make this collection uniquely useful for the characterization of host-microbe, microbe-microbe, and microbe-environment interactions in the skin microbiome.

Our study primarily focused on bacterial isolates. These organisms represent 25 families and ~ 38 genera. Additionally, we collected 24 fungal isolates representing 14 genera. Many of these organisms have been described in previous studies, but some unique taxa were identified. An advantage of this study is the inclusion of distinct isolation conditions, shown in Table 1 with results summarized in Fig. 1 and Additional file 1 Table S8. With the caveat that these conditions were not applied to all 17 subjects (see Table 1), and in some cases only 1 subject, we investigated the ability of these methods to increase cultural diversity. We observed that room temperature blood agar, anaerobic culturing on blood agar, R2A media, and plating results of repeated tape stripping resulted in a diverse collection of organisms. This diversity is likely due to culturing of the anaerobic fraction, accommodation of slow growers using low nutrient environment (R2A), and access to potentially distinct fractions of the skin microbiome by successive tape stripping. Future studies should carefully consider culturing conditions for the isolation of representative populations. In addition, an interesting consideration for skin microbiome studies is the role of transient strains. While some of our isolates may be more commonly considered soil or environmental organisms, they nonetheless can be found on the skin of healthy individuals. If and how these organisms contribute to the function of the skin microbiome is unknown and is a meaningful area of investigation for future research.

**Table 1:** Culture conditions for isolating organisms

Target	Media	Temperature	Special conditions	Leading number in isolate ID ^1^	Research participant(s)
Aerobes	Blood agar (Hardy Diagnostics)	Room temp.	Aerobic	0	All
Aerobes	Blood agar (Hardy Diagnostics)	37 °C	Aerobic	1	60, 63, 66, 70
Anaerobes	Blood agar (Hardy Diagnostics)	37 °C	Anaerobic^3^	2	60, 63, 66, 70
Slow growers	R2A (BD Biosciences)	Room temp.	Aerobic	4	63, 66, 70
Lactobacilli	MRS (Difco)	Room temp.	Aerobic	6	63, 66, 70
Fungi	PDA (BD Biosciences)	Room temp.	Aerobic	5	63, 66, 70
Spores	Blood agar (Hardy Diagnostics)	Room temp.	4 h incubation in 70% EtOH, aerobic	7	63, 66, 70
Anaerobic spores	Blood agar (Hardy Diagnostics)	37 °C	4 h incubation in 70% EtOH, anaerobic^3^	8	66, 70
Capnophiles	Blood agar (Hardy Diagnostics)	37 °C	Aerobic + 5% CO2	3	60
Gram-negative	MacConkey	Room temp.	Aerobic, dark	6	70
Halophiles	Medium for halophilic bacteria^2^	Room temp.	10% NaCl, aerobic	3	70
Anaerobic halophiles	Medium for halophilic bacteria^2^	37 °C	10% NaCl, anaerobic^3^	9	70
Deep skin aerobes	Blood agar (Hardy Diagnostics)	37 °C	Tape strips #5 and #10, aerobic	TS5, TS10	70
Deep skin anaerobes	Blood agar (Hardy Diagnostics)	37 °C	Tape strips #5 and #10, anaerobic^3^	TS5a, TS10a	70

^1^For example, FM060_X01 represents the first isolate from the forehead site (FM) from research participants # 60, where *X* is the leading number

^2^DSMZ 73 media, 10 g/L casamino acids (Sigma), 10 g/L yeast extract (Sigma), 100 g/L NaCl, pH7.0 [37]

^3^Anaerobic conditions were obtained using the BD GasPak EZ system (Fisher Scientific)

We screened a subset of representative, aerobic, isolates for the ability to utilize carbon sources present on the skin surface using tetrazolium dye assays with custom carbon sources. The 25 isolates characterized by this assay included *Firmicutes*, *Actinobacteria*, and *Proteobacteria* representatives, and metabolism generally clustered with phylogenetic relationships of strains. Lacking from the group are *Cutibacterium* representatives (formerly *Propionibacterim*), which play an important role in metabolite processing on the skin [11]. The strains tested utilized a total of 45 out of the 68 carbon compounds examined, which included steroids, fatty acids, sugars, amino acids, and other classes of chemicals. These results indicate that isolated organisms have the ability to metabolize a variety of compounds found in the skin environment. A range of specialists and generalists was observed, and the diversity of utilization patterns may play a role in the observed coexistence of diverse taxa in the skin environment. We found different patterns in resource utilization even across bacteria of the same genera, underscoring the need to consider species and strain level differences in microorganism function. Despite the differences observed at the species and strain levels, phylogenetic distance analysis showed that there was a significant and positive relationship between the phylogenetic similarity of microbial taxa and the carbon sources they utilized. This finding was strongest among generalist taxa (those that utilized four or more carbon sources), with more phylogenetically similar taxa utilizing increasingly overlapping sets of carbon sources.

Collectively, these results suggest that there is some degree of validity to inferring community properties from phylogenetic characterization [33], yet strain level information can be critical to obtaining an accurately detailed profile. In addition, recent work by Bouslimani et al. [10] has revealed that skin hygiene products can remain on the skin long after use is discontinued, and these products can influence microbiome composition [11]. Our approach may be used to develop a more detailed understanding of how and why the microbiome shifts in response to the use of certain hygiene and cosmetic products. Towards this direction and other in vitro characterization efforts, the size and diversity of the presented isolates collection make it a powerful resource for skin microbiome research.

In gut microbiome research, a synergistic interplay between culturing, sequencing, and informatics approaches is exemplified by the identification of a “most wanted” list of microbes [34], followed by targeted culturing efforts which concluded that most gut microbes are likely culturable [35]. Along similar lines, we envision a growing convergence of approaches that assimilates sequencing and informatics, microbial trait characterization [36], and in vitro studies with cultured isolates, which will deepen our understanding of the skin microbiome composition, its role in health, and ultimately methods for optimizing it.

## Conclusion

Until now, the majority of available commensal skin microorganisms were from the Human Microbiome Project (HMP) which provided valuable insight into human microbiome interactions. With this publicly available collection (BEI Resources, with additional strains available by correspondence), we increased the number of skin commensal genera available (Additional file 2 Figure S1). Additionally, we added significant diversity to the *Staphylococcus* and *Micrococcus* genera. Our collection also includes 12 *Paenibacillus*, 8 *Roseomonas*, and 7 *Dietzia* isolates that were previously not cataloged by the HMP. The increase in diversity in our collection relative to other reports is likely due to increased throughput. Specifically, the most abundant strains in our collection (*Staphylococcus*, *Micrococcus*, *Bacillus*, *C. acnes*) are representative of amplicon studies and other culture collections from the skin microbiome, while the rarer genera in our collection have been described in distinct cases. We believe this collection is a resource that will greatly increase our understanding of the skin microbiome through further genomic characterization and analysis, in vitro experiments, and synthetic biology endeavors.

## Methods

### Human research participants

Human research participants were sampled at Johns Hopkins Bayview Medical Center according to a protocol approved by the Johns Hopkins and the U.S. Army Human Research Protection Office Institutional Review Boards. The purpose of this study was to generate microbial ecology data for the skin microbiome. Participants were healthy volunteers, aged 18–50, with no history of chronic skin conditions or autoimmune diseases. Research participants were asked to not shower/bathe 2 days prior to sampling and answered a 400-point questionnaire (Additional file 1 Table S1) and then two swabs from each site were collected. To quantify skin properties, commercially available probes were used adjacent to swab sites in conjunction with the Multi Probe Adapter 10 system (Courage and Khazaka, GmbH) according to manufacturer instructions. Specifically, a Sebumeter was used to quantify sebum content and a Tewameter TM300 was used to quantify transepidermal water loss. Metadata for each volunteer is included in Additional file 1 Table S1 and summarized briefly in Fig. 1. Isolates data were not compared to subject metadata. There was no clinical intervention in this study for which to group volunteers and perform additional analyses.

### Sample plating, growth conditions, and isolations

Healthy human research participants were sampled from the forehead, forearm, or antecubital fossa (inner elbow) by swabbing with cotton swabs saturated in 50 mM Tris (Amresco), 1 mM EDTA (Amresco), 0.5% Tween 20 (Sigma) in nuclease-free water for 30 s. For all participants, swabs were plated immediately on blood agar (Hardy Diagnostics) and incubated until colonies were observed. For four selected individuals, a second adjacent swab from the same site was added to 4 ml sterile TSB and mixed to resuspend bacterial samples for further plating, as described in Table 1.

From each plate, all phenotypically distinct colonies were picked onto fresh media for isolation. Single colonies were picked and re-streaked at least three times to isolate individual strains. Strains were grown in TSB, DSMZ 73 or on plates under corresponding aerobic/anaerobic and temperature conditions and frozen at – 80 °C in 25% glycerol. A subset of representative isolates (Additional file 1 Table S2) has been deposited at BEI resources (https://www.beiresources.org) for curation and distribution to the scientific community.

### Isolate identification and 16S gene analysis

Aliquots of glycerol stocks were processed at Genewiz LLC (South Plainfield, NJ, USA) by colony PCR of the full 16S rRNA gene and subsequent Sanger sequencing. Resulting forward and reverse reads were merged using the EMBOSS merger [38], and then merged reads were aligned using the assignment and classification functions at SINA [39] to available databases using default parameters (Additional file 1 Table S3). Low quality and unmerged reads were manually curated, and then forward and reverse reads were classified using BLAST.

### Generation of 16S and ITS phylogenetic trees

In addition to the 16S sequences from isolates, 16S sequences from 83 reference strains were added to help speciate isolate groups (Additional file 1 Table S4). To facilitate tree generation, 16S sequences that did not contain at least 1280 unambiguous nucleotides were removed. The remaining sequences were then aligned in a multiple sequence alignment using MAFFT (version 7.123b) [40] along with *Escherichia coli K-12* for identification of the variable regions. All sequences were then trimmed to the variable regions V2–V8 using the *E. coli* reference [41–43] to normalize due to quality issues in some V1 regions. These truncated 16S sequences (739 sequences) were analyzed using BLAST with the SILVA SEED database (release 132), and the full-length 16S sequence from the top BLAST hit for each isolate was used as reference sequences (95 total). Reference identity was taken from the corresponding SILVA entry except for the asterisked sequences which were classified using the SILVA classification service [39]. The isolates and reference sequences were aligned and trimmed to the V2–V8 region using the default settings of MAFFT (version 7.123b) [40]. Isolates with more than 5 unidentifiable bases (.5%) within this region were not included in the tree (46 sequences). Sequences were realigned with Infernal (1.1.2) [44] using the bacterial SSU rRNA covariance matrix downloaded from Rfam. Initial tree making was run with FastTree (version 2.1.7). Tree refinement was run with RAxML (version 8.2.0), first using the rapid hill-climbing method with the GTRCAT substitution model with bootstrapping. Further refinement was run with the RAxML [45] model and branch length optimization method using the GAMMA substitution model. Trees were visualized using the r2d3 package in R.

### Fungal isolates

Molds were identified by tissue growth on plates and maintained by plug passaging, and yeasts were identified by microscopy and maintained as streak cultures. gDNA was extracted from fungal samples using the DNEasy UltraClean Microbial DNA isolation kit (QIAGEN) using 4x bead-beating/freeze-thaw cycles to lyse cells: flash freeze in liquid nitrogen, heat to 65 °C, and bead beat in the TissueLyser II for 10 min at 25 Hz. For yeasts, lysing was replaced by colony PCR. Internal transcribed spacer (ITS) regions were amplified using published primers F-5′-GTAAAAGTCGTAACAAGGTTTC and R-5′-GTTCAAAGAYTCGATGATTCAC ( ITS1) and F-5′-GTGAATCATCGARTCTTTGAAC and R-5′-TATGCTTAAGTTCAGCGGGTA (ITS2) [46]. PCR products were purified using magnetic beads then sequenced at Genewiz using the forward and reverse primers. Reads were merged using the EMBOSS merger [38], and then the top hit from BLAST analysis was used to determine putative species. Identification of fungal strains is included in Table S4.

### Preparation of skin compound utilization assay

Skin-relevant compounds were selected based on a literature search for compounds detected in sweat, sebum, and as residual skin surface chemicals. All skin-relevant compounds assessed, their sources and literature sources citing their presence on the skin are listed in Additional file 1 Table S6. To prepare the assay plates, compounds were dissolved in molecular biology-grade water or chloroform (Fisher Scientific), at 10 mg/ml. Stock solutions were distributed into polypropylene 96-well plates. Negative controls consisted of three wells containing only water and three wells containing only chloroform. A positive growth control consisted of three wells with 10% TSB in water. Water and chloroform were evaporated so that assay plates contained only 0.3 mg of a single carbon source per well. Plates were stored covered at 4 °C until use.

### Bacteria culturing and preparation of assay inoculation cultures

Bacteria were stored in 10% glycerol stocks at – 80 °C. To generate starter cultures, glycerol stocks were streaked individually on TSA plates and incubated at 30 °C. Single colonies were picked to inoculate 5 ml of TSB, which was incubated at 30 °C with shaking for 1 to 3 days. To prepare the assay inoculation culture, starter cultures were diluted ~1:500 and cultured for approximately 4 h in TSB. Bacteria pellets were washed three time by centrifugation at 4300 G for 10 min, aspiration of the supernatant, and gentle resuspension in an essential salt solution adapted from Bochner et al. [47] consisting of 100 mM sodium chloride, 30 mM triethanolamine, 25 mM sodium pyruvate, 5.0 mM ammonium chloride, 2.0 mM monosodium phosphate, 0.25 mM sodium sulfate, 0.05 mM magnesium chloride, 1.0 mM potassium chloride, and 1.0 μM ferric chloride (all reagents were purchased from Sigma). The optical density at 600 nm (OD) of the final bacterial solution was measured using a Nanodrop 2000c spectrophotometer (Thermo Scientific) and bacteria were brought to an assay OD of 0.001 supplemented with Biolog dye mix A (Biolog, Inc.) to achieve a 1× concentration immediately before the assay.

### Assay for skin compound utilization

Bacterial solution (0.2 ml) was added to each well of the assay plate, and sterile water was introduced in the spaces between wells to increase local humidity. Plates were incubated at 30 °C in a humidity chamber without shaking. Assessment of compound utilization was measured at OD at 590 nm using a CLARIOstar plate reader (BMG Labtech) immediately after plate preparation and 72 h later. At least three assay plates were examined for each bacterial isolate. Absorbance values from the 0 time point were subtracted from the 72 h values to yield background subtracted values. Compound utilization was then assessed by an ANOVA followed by a Dunnett’s test against the negative control with a cut-off *p* value of 0.05 using JMP® (Version 13.0.0, SAS Institute Inc., Cary, NC, 1989–2019). If positive control wells did not show growth for a bacterial isolate, the bacterial concentration was increased 10-fold up to two times. Each set of assays included a plate with the essential salt solution and Biolog dye mix A without a bacterial inoculation to ensure sterility of the assay plate. Compounds were classified using the BioCyc database [48], where the most specific parent class was chosen that allowed for molecule classifications with three or more compounds. Compounds not in the BioCyc database were classified with the ClassyFire tool [49]. Any compounds unable to be categorized by either tool are grouped into the ‘miscellaneous’ category.

### Phylogenetic distance analysis

Carbon source utilization was compared to the phylogenetic similarity of microbial taxa using two approaches: (i) Mantel tests and (ii) linear regression models. For both approaches, phylogenetic similarity was measured using phylogenetic distances calculated from the trimmed V2–V8 16S rRNA sequences described above and the similarity in carbon source utilization was measured using the Jaccard index. For a given pair of microbial taxa, the Jaccard index equaled 1 if the two microbial taxa utilized the same set of carbon sources, 0 if they utilized completely different carbon sources, and values between 0 and 1 depending on the proportional overlap of carbon sources. A Mantel test returns the correlation between two matrices, in this case, between a square matrix of phylogenetic similarity values (each element was the phylogenetic similarity of a pair of microbial taxa) and a corresponding square matrix of Jaccard similarity values (each element was the Jaccard index for a pair of microbial taxa). With linear regression models, Jaccard similarity was used as the response variable and phylogenetic similarity as the predictor variable. Because most pairs of microbial taxa did not share any carbon sources, linear regression models were also fit but excluding pairs of microbial taxa that did not share at least one carbon source.

## Supplementary information

**Additional file 1.** This file contains supporting tables and information not included in the primary text.

**Additional file 2: Figure S1.** Increase in available skin microbiome representatives by this project.

## Data Availability

Data is available in online supplemental information. Microbial strains are available from BEI Resources (see Table S2) or from authors upon request.
