# Supplementary figures and images for: Isolation and characterization of diverse microbial representatives from the human skin microbiome
Source: Microbiome. 2020 Apr 22;8:58. doi: 10.1186/s40168-020-00831-y (PMC7178971; doi:10.1186/s40168-020-00831-y)

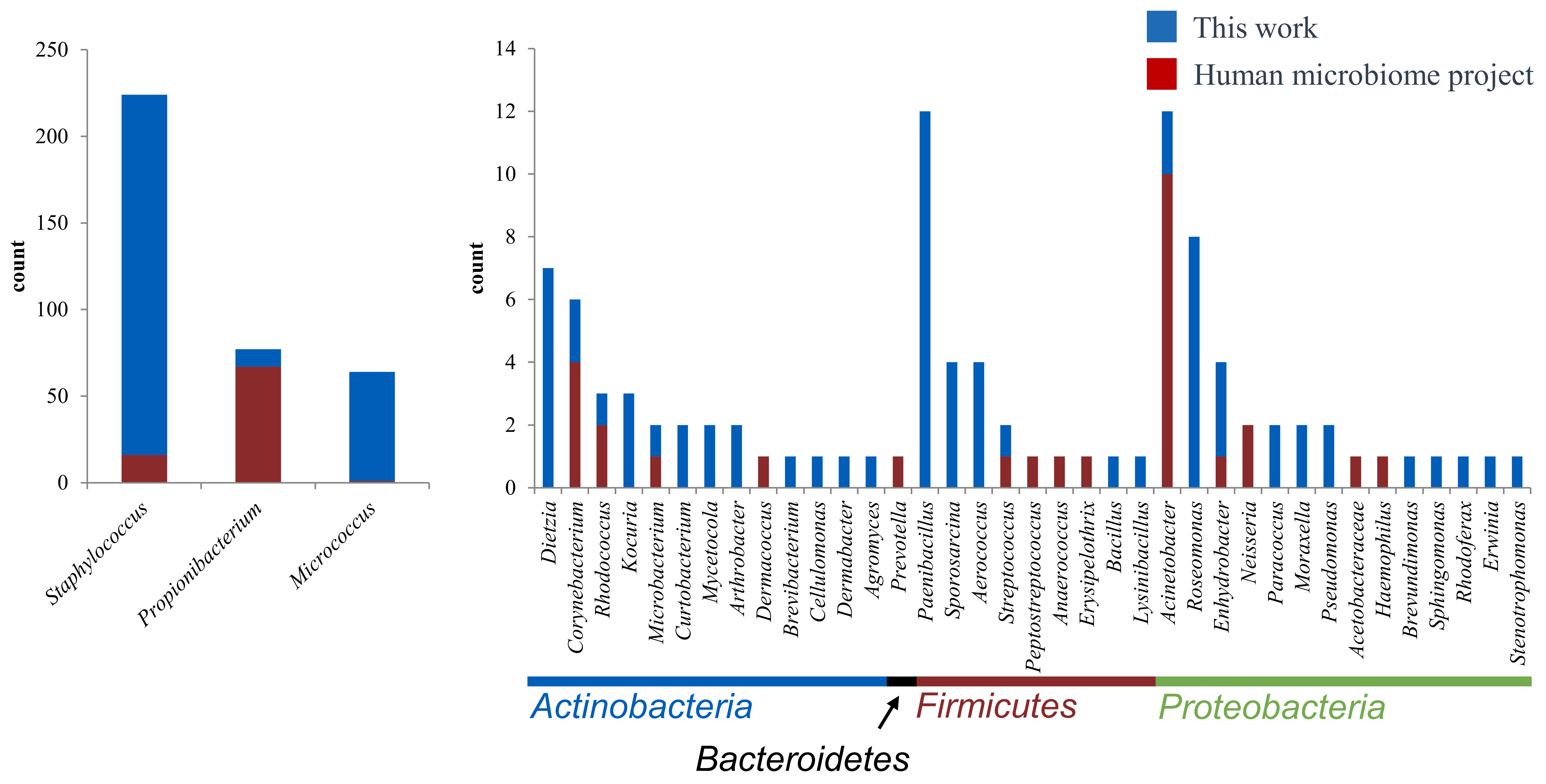

Supplement: Supplementary file 3 — Additional file 2: Figure S1. Increase in available skin microbiome representatives by this project. [file 40168_2020_831_MOESM2_ESM.tif]
